# FROM MODEL ORGANISMS TO HUMANS, THE OPPORTUNITY FOR MORE RIGOR IN METHODOLOGIC AND STATISTICAL ANALYSIS, DESIGN, AND INTERPRETATION OF AGING AND SENESCENCE RESEARCH

**DOI:** 10.1093/geroni/igad104.0064

**Published:** 2023-12-21

**Authors:** Steven Austad

**Affiliations:** University of Alabama at Birmingham, Birmingham, Alabama, United States

## Abstract

DOI: 10.1093/gerona/glab382

